# Intrapartum Ultrasound in Vacuum Operative Delivery: A Comprehensive Review and Proposal of the Novel Ultrasound Flexion Point Method

**DOI:** 10.3390/diagnostics16060946

**Published:** 2026-03-23

**Authors:** Antonio Malvasi, Giorgio Maria Baldini, Tommaso Difonzo, Marco Cerbone, Iris Cara, Marianna Demarco, Ilenia Mappa, Giuseppe Rizzo, Antonella Vimercati, Miriam Dellino, Andrea Tinelli, Edoardo Di Naro, Lorenzo E. Malgieri

**Affiliations:** 1 Obstetrics and Gynecology Unit, Department of Interdisciplinary Medicine (DIM), University of Bari “Aldo Moro”, 70124 Bari, Italy; antoniomalvasi@gmail.com (A.M.); difonzo.tommaso.md@gmail.com (T.D.); marcocerbone@gmail.com (M.C.); carairis95@gmail.com (I.C.); antonellavimercati@gmail.com (A.V.); miriamdellino@hotmail.it (M.D.); dinaroedoardo@gmail.com (E.D.N.); 2 Medical School, University of Bari, 70124 Bari, Italy; 3 Department of Maternal and Child Health and Urological Sciences, University of Roma Sapienza, 00185 Rome, Italy; mappa.ile@gmail.com (I.M.); giuseppe.rizzo@uniroma1.it (G.R.); 4 Department of Obstetrics and Gynecology and CERICSAL (CEntro di RIcerca Clinico SALentino), Veris Delli Ponti Hospital Scorrano, 73020 Lecce, Italy; andreatinelli@gmail.com; 5 The New European Surgical Academy (NESA), 10117 Berlin, Germany; lorenzo@malgieri.org

**Keywords:** vacuum delivery, intrapartum ultrasound, flexion point, cup placement, fetal complication, vacuum failure, vacuum detachment, operative vaginal delivery, midline angle, angle of progression, biofeedback

## Abstract

Operative vaginal delivery (OVD) via vacuum extraction is a fundamental component of modern obstetric management, yet it carries specific risks of failure and maternal–fetal complications, such as cup detachment, cephalohematoma, and intracranial hemorrhage. The success and safety of the procedure rely heavily on the correct application of the vacuum cup over the “flexion point” of the fetal head. Traditional identification of this landmark via digital examination is often hindered by caput succedaneum and cranial molding, leading to high rates of diagnostic error, particularly in dystocic labor, due to fetal head malpositions and malpresentation. Intrapartum ultrasound (ITU) has demonstrated superior accuracy compared to clinical examination in assessing fetal head position and station and internal rotation. This expert commentary and technical proposal analyzes the current literature regarding vacuum extraction application and failures, focusing on the predictive value of ITU parameters (e.g., Angle of Progression, Midline Angle, Head-Symphysis Distance) and the impact of ITU on cup placement and delivery outcomes. Furthermore, we propose a novel technique: the “Ultrasound Flexion Point” (UFP). This method utilizes translabial ultrasound to identify the specific intersection of the fetal midline and the biparietal diameter as an objective sonographic proxy for the classical flexion point. By providing spatial orientation guidance immediately before the procedure, this technique aims to guide the operator in aligning the cup’s notch with the sonographically identified target zone, using the midline angle as orientation reference, thereby potentially minimizing paramedian or deflexing applications and reducing the incidence of vacuum detachment and associated neonatal trauma. This expert commentary and technical proposal synthesizes current evidence and proposes a protocol requiring prospective validation through randomized controlled trials.

## 1. Introduction

Operative vaginal delivery (OVD) remains a cornerstone of modern obstetric management, providing a vital alternative to cesarean section in the second stage of labor. It is defined as the expedited delivery of the fetus using instruments such as forceps or a vacuum extractor when indicated by specific maternal or fetal conditions. Despite the rising rates of cesarean deliveries globally, OVD continues to represent a significant proportion of births, accounting for approximately 3.3% of all deliveries in the United States in 2013 [1]. The choice of instrument often depends on the clinical scenario and the operator’s expertise. Among the available tools, the vacuum extractor has gained popularity due to its lower risk of maternal perineal trauma compared to forceps [2]. Specifically, the OmniCup vacuum device, designed by Aldo Vacca, has become a widely used instrument in obstetric practice due to its versatility and design features intended to facilitate correct application [3,4]. Recent literature has extensively compared the safety and efficacy of different vacuum devices. Evidence suggests that the Kiwi OmniCup system can reduce the incidence of severe neonatal morbidity without increasing serious adverse maternal outcomes compared to other devices [5]. Similarly, Turkmen et al. demonstrated that both the Kiwi OmniCup and Malmström metal cup are functionally effective and safe [6]. However, the type of cup used can influence the procedural success rate. For instance, Equy et al. reported in a multicenter randomized controlled trial that disposable cups were associated with higher rates of detachments and extraction failures compared to Drapier-Faure metallic cup, although they resulted in fewer perineal injuries [7]. Groom et al. corroborated these findings, noting that the Kiwi OmniCup was less successful as an instrument of first choice compared to conventional vacuum cup (failure rate 30.1% vs. 19.2%; RR 1.58) and was associated with a significantly higher number of cup detachments [8].

Despite this, the safety profile regarding severe maternal or neonatal trauma remained comparable between the devices.

The success of vacuum extraction is intrinsically linked to the biomechanics of the procedure, specifically the site of cup application. The “flexion point” is defined as a pivot point on the fetal skull, located along the sagittal suture approximately 3 cm anterior to the posterior fontanelle and 6 cm posterior to the anterior fontanelle. When the center of the vacuum cup is correctly placed over this point, traction promotes flexion of the fetal head, minimizing the presenting diameters and reducing resistance from the birth canal and pelvic floor [9]. Proper flexion is crucial as it is associated with lower resistance during descent and reduced stress on the pelvic floor [10]. Conversely, deflexion or paramedian application leads to asynclitism and larger presenting diameters, significantly increasing the risk of failure and complications.

Assessing fetal head position and flexion is therefore paramount. While digital vaginal examination (DE) is the traditional standard, its accuracy is often compromised, particularly in the presence of caput succedaneum or significant molding, which can mask sutures and fontanelles. Akmal et al. demonstrated that intrapartum digital examination fails to identify the correct fetal head position in approximately one-quarter of cases, with accuracy dropping to 54% for occiput-lateral and occiput-posterior positions [11]. Incorrect diagnosis of head position is a leading cause of vacuum application errors. Kong et al. highlighted that vacuum failure generally stems from incorrect cup placement [12]. Furthermore, misplacement can lead to an imbalance of tension forces on the tentorium cerebelli, potentially causing intracranial hemorrhage and other severe birth traumas [13,14].

The consequences of suboptimal application are not trivial. A retrospective cohort study by Schreiber et al. identified that cup detachment (pop-off) is associated with adverse outcomes, including higher rates of subgaleal hematoma (8.9% vs. 3.5%), lower cord blood pH (<7.15), and increased Neonatal Intensive Care Unit (NICU) admissions [15]. These complications are frequently linked to identifiable risk factors such as occiput posterior position, macrosomia, and prolonged vacuum duration.

Given the limitations of digital examination, Intrapartum Ultrasound (ITU) has emerged as a superior diagnostic tool. Several authors have confirmed that ITU provides greater accuracy in determining fetal head position and station during the second stage of labor [16,17,18,19].

Specific sonographic parameters have demonstrated significant predictive value for vacuum delivery outcomes. The Angle of Progression (AoP) has been extensively studied as a prognostic marker. In 2016, Sainz et al. identified that an AoP < 105° measured during maternal pushing, combined with a progression distance < 25 mm and a midline angle > 45°, represents a constellation of very unfavorable parameters associated with high risk of extraction failure [20]. In 2015, Bultez et al. further demonstrated that AoP is a predictive factor for failed vacuum extraction, particularly among nulliparous women whose baseline risk of failure is elevated [21]. The predictive utility of head–perineum distance (HPD) has been quantified by Kahrs et al. in a multicenter prospective cohort study; women with HPD ≤ 25 mm experienced significantly shorter extraction duration (median 6.0 vs. 8.0 min, *p* < 0.01) and lower cesarean conversion rates (3.9% vs. 22.0%) compared to those with HPD > 35 mm [22]. Kasbaoui et al. refined these distance thresholds, demonstrating that a perineum-to-skull ultrasound distance ≥ 40 mm was significantly associated with difficult extraction, establishing this as a reproducible and predictive index of procedural complexity [23].

A comprehensive meta-analysis by Nassr et al. confirmed that AoP measured during the second stage of labor is a reliable predictor of uncomplicated operative vaginal delivery, with particularly high sensitivity and specificity in nulliparous women [24]. More recently, Skinner et al. conducted a comparative systematic review and meta-analysis evaluating the prognostic accuracy of multiple intrapartum transperineal ultrasound measures [25]. They demonstrated that angle of progression, progression distance, and midline angle measured with maternal pushing exhibit the highest prognostic accuracy in predicting complicated or failed operative vaginal birth, with moderate accuracy for head direction and head–perineum distance, and fair accuracy for midline angle alone [25].

However, while systematic reviews indicate that ITU reduces incorrect diagnoses of fetal head position and station [26], this improved diagnostic accuracy has not yet consistently translated into improved maternal or neonatal outcomes in all studies. Bellussi et al. in 2022 conducted a systematic review and meta-analysis of randomized controlled trials in which they concluded that sonographic knowledge of occiput position before operative vaginal delivery does not appear to reduce the incidence of failed procedures despite superior diagnostic accuracy compared to clinical examination alone [27]. This paradox suggests that diagnosis alone is insufficient; the sonographic information must be actively integrated into the procedural technique itself to translate diagnostic precision into improved clinical outcomes.

## 2. Materials and Methods

This manuscript is structured as an expert commentary and technical proposal. We synthesized current evidence focusing specifically on operative vaginal birth, intrapartum ultrasound, and the biomechanics of cup placement, to provide the rationale for a novel ultrasound-guided orientation method. General guidelines for operative vaginal delivery are briefly summarized, focusing strictly on factors influencing placement accuracy.

Across international guidance, prerequisites for vacuum-assisted birth are consistent: full cervical dilatation, ruptured membranes, engaged cephalic presentation with the head at or below the ischial spines, accurate assessment of fetal head position, an empty maternal bladder, adequate analgesia, informed consent, and immediate capability for escalation (including cesarean delivery) if the attempt fails [1,28,29,30]. Similar prerequisites are reiterated in Canadian (Society of Obstetricians and Gynaecologists of Canada [SOGC]) and Central European (DGGG/ÖGGG/SGGG) guidance and in National Institute for Health and Care Excellence (NICE) intrapartum care recommendations that address operative vaginal birth within broader intrapartum management [31,32,33] (Table 1).

Major international guidance is broadly concordant on prerequisites, contraindications, and early abandonment criteria for vacuum-assisted birth, including the American College of Obstetricians and Gynecologists (ACOG) Practice Bulletin [1,34], the Royal College of Obstetricians and Gynaecologists (RCOG) Green-top Guideline No. 26 [28], the International Federation of Gynecology and Obstetrics (FIGO) good practice recommendations [30], the Royal Australian and New Zealand College of Obstetricians and Gynaecologists (RANZCOG) statement on instrumental vaginal birth [29], the Society of Obstetricians and Gynaecologists of Canada (SOGC) clinical practice guideline [31], the DGGG/ÖGGG/SGGG guideline on vaginal-operative birth [32], and contemporary national guidance emphasizing standardized documentation and ultrasound use when clinical uncertainty exists [33].

**Table 1:** Commonly cited early reassessment triggers and discontinuation thresholds (pulls, cup detachments, and time) for vacuum-assisted birth, as reported in selected international guidance. Numeric thresholds are operational triggers and should be interpreted in the context of progressive descent, operator expertise, and maternal–fetal status. Numeric thresholds vary across guidance and should be applied with clinical judgement, continuous reassessment of descent and fetal status, and local governance. Across documents, early reassessment and timely senior input are emphasized.

Organization (Year)	Early Reassessment Trigger	Max Traction Attempts (Pulls)	Cup Detachments (Pop-Offs)	Time Limit (Minutes)	Key Clinical Notes
RCOG (2020) [28]	No descent with the first 2 pulls by an experienced operator.	Abandon if no birth after 3 pulls; max 3 pulls for descent + 3 to deliver.	Abandon if the cup detaches for a 3rd time (max 2).	Should not exceed 15–20 min.	Emphasizes “Trial of Vacuum” in theatre for mid-cavity cases.
FIGO (2025) [30]	If no descent after 2 pulls.	Max 3 pulls for descent; additional pulls only if crowning is imminent	Stop if the cup dislodges twice (max 2).	20 min maximum.	Pop-offs are often due to incorrect axis or cephalopelvic disproportion.
ACOG (2020) [1]	No descent with the “first several pulls” (not numeric).	No evidence-based limit; suggests reassessment if birth is not imminent.	No evidence-based numeric limit stated.	No specific time limit provided.	Focus on “Flexion Point” placement (3 cm anterior to posterior fontanelle).
RANZCOG (2020) [29]	If birth not imminent after 15 min.	Max 3 pulls without descent of the skull (not scalp).	Up to 3 acceptable; re-apply only if there is progress or head on perineum.	Suggested upper limit: 20 min.	Cites Vacca; notes SGH risk from rapid decompression after pop-off.
DGGG/ÖGGG/SGGG (2025) [32]	If birth not imminent after ~3 contractions.	Aim to complete in 3 tractions; reassess if no descent/imminent delivery.	No numeric limit; notes that detachments increase neonatal morbidity.	No evidence-based threshold for traction-to-delivery	Recommends ultrasound if digital exam is uncertain.
FEBRASGO (2023) [35]	Stop if no evidence of progressive descent.	Usually up to 3 pulls; 3 additional gentle pulls to complete deflection.	Stop if cup detaches on 3 occasions.	Stop if traction time exceeds 20 min.	Recommends avoiding vigorous movements during application.

Table note: Summary derived from international guidance and selected national guidance. Where numeric thresholds are suggested, they are presented as operational triggers rather than universal physiological cut-offs; continuous assessment of descent and maternal–fetal status is essential. The anatomical flexion point cited by ACOG (approximately 3 cm anterior to the posterior fontanelle) represents the classic digital landmark. The Ultrasound Flexion Point (UFP) proposed in this manuscript is a sonographic proxy intended to approximate this target zone when digital palpation is unreliable; it is not claimed to be anatomically identical to this landmark.

Contraindications and cautionary contexts also converge. Vacuum extraction is generally discouraged for preterm birth below 34 weeks’ gestation and used with caution at 34–36 weeks, because of higher susceptibility to scalp and intracranial injury [1,30]. Several national statements also recommend avoidance at very early gestations and emphasize careful counseling and neonatal readiness when vacuum is used at late preterm gestations [35].

Stop rules (often framed as ‘safety stops’) are central to safe practice. Guidelines emphasize abandoning the attempt when there is inadequate descent despite correctly directed traction, when detachments recur, when the number of pulls exceeds local limits, or when maternal or fetal conditions change such that continuation is unsafe [1,28,30]. Detachment should be treated as a safety signal rather than an expected event.

Thresholds for discontinuation varied: while RCOG [28] and FIGO [30] provide strict numeric limits (e.g., 2–3 detachments or 15–20 min), ACOG [1] emphasizes clinical reappraisal without fixed numeric triggers. All guidelines converge on the necessity of abandoning the procedure if progressive descent is not achieved within the first two to three pulls. Guidance also cautions against routine sequential instrumentation because it increases neonatal risk; if sequential instruments are considered, the rationale should be explicit and the risks reassessed in real time [30,35].

To support clinical practice and safety, we propose a practical checklist for vacuum-assisted birth (Table 2) and a standardized documentation dataset (Table 3), derived from the synthesis of international recommendations.

This checklist is intended as a cognitive aid and local documentation minimum dataset; units should adapt it to local governance and documentation systems.

### The “Ultrasound Flexion Point” Method: A New Proposal

Based on the analysis of the literature identifying “incorrect cup placement” as the primary cause of vacuum failure and fetal trauma [12,13], we propose a novel ultrasound orientation method. This method integrates intrapartum ultrasound (ITU) directly before the vacuum application procedure to objectively identify the flexion point, especially in cases with the presence of caput succedaneum and molding.

The proposed technique consists of a sequential approach involving transabdominal and translabial assessments, followed by ultrasound application.

Step 1: Preliminary Transabdominal Assessment. Before instrument application, a transabdominal ultrasound scan is performed to confirm the fetal head position (occiput anterior, posterior, or transverse) and the position of the fetal spine. This step rules out gross malpresentations that would contraindicate vaginal delivery.

Step 2: Translabial Geometric Assessment. A convex probe is placed translabially to collect four geometric parameters in two planes. First, in the mid-sagittal plane (parallel to the infrapubic line), the Angle of Progression (AoP) and the Head–Symphysis Distance (HSD) are measured during active maternal pushing. Second, in the transverse plane (parallel to the interspinous line), the Midline Angle (MLA) and the degree of asynclitism are assessed. These prognostic parameters are used to evaluate procedural feasibility:

- Midline Angle (MLA): To quantify the degree of head rotation. An MLA > 45° may indicate malrotation [20].
- Progression Indices: Measurement of the Angle of Progression (AoP) [22,24] and Head–Symphysis Distance (HSD). Using a mid-sagittal translabial view, these parameters must be measured during active maternal pushing. In our protocol, we exclusively utilize HSD (<35 mm). As detailed in our previous works on the Artificial Intelligence Dystocia Algorithm (AIDA) [36,37,38], we prefer HSD over the Head–Perineum Distance (HPD) because it relies on a fixed, rigid bony landmark (the symphysis pubis). This avoids the inherent variability of soft-tissue metrics like HPD, which are heavily influenced by perineal distensibility and edema. Similarly, AoP and MLA achieve their highest prognostic accuracy when measured during active maternal pushing [20,24,25].
- Asynclitism Degree: Evaluation of the symmetry of the cerebral structures. A minimal asynclitism is operationally defined as a midline that remains relatively central with symmetric visualization of the thalami, whereas severe lateral deviation predisposes to tentorial tears [37,38]. The asynclitism degree (AD) is measured on the same transverse translabial view used for MLA assessment. In a synclitic presentation, the midline lies equidistant between the two parietal bones; in asynclitism, it deviates laterally—toward the pubis in posterior asynclitism or toward the sacrum in anterior asynclitism [37,38]. The AD is quantified as the perpendicular distance (in mm) between the midline and the nearest parietal bone, as described and validated in our AIDA studies [36,37,38]. Based on decision-tree analysis of 135 cases, AD < 65.5 mm was associated with non-cesarean delivery (green zone), AD ≥ 70.5 mm was strongly predictive of intrapartum cesarean delivery (red zone), and a transitional yellow zone (65.5–70.5 mm) was identified between these thresholds [36,37,38]. In the present protocol, we operationally define “minimal asynclitism” as AD within the green zone, corresponding to a midline that remains relatively central with symmetric thalamic visualization. In our algorithm, this assessment serves as a qualitative screening step (i.e., “minimal vs. severe”) rather than a quantitative substitute for a continuous asynclitism severity scale, which has not yet been validated sonographically. Cases judged as “severe” trigger escalation or enhanced caution, whereas “minimal” asynclitism permits the operator to proceed with vacuum application.

Step 3: Identification of the “Ultrasound Flexion Point” (UFP). This is the core of the new method. In the transverse translabial view:

1. The Fetal Midline (representing the sagittal suture) is visualized.
2. The Biparietal Diameter (BPD) is identified.
3. The image is frozen.
4. The intersection point between the Midline and the BPD is identified.

We define this intersection as the “Ultrasound Flexion Point”. While not anatomically identical to the classic flexion point (located ~3 cm anterior to the posterior fontanelle, which is often obscured by caput succedaneum), this sonographic intersection serves as a reliable, pragmatic proxy. We acknowledge that obtaining a perfect transverse BPD plane can be challenging due to probe angle, severe molding, or a crowded perineum. In such cases, the operator should aim for the most symmetrical view of cerebral structures (e.g., thalami) to define the midline and then identify the UFP at the midpoint of the midline, using this map as a spatial target when tactile landmarks fail.

Step 4: Biofeedback-Guided Application and Cognitive Mapping. It is crucial to clarify that neither the vacuum cup (e.g., Kiwi OmniCup) nor its indicator notch is visible on ultrasound. Therefore, the frozen transverse ultrasound image serves purely as a spatial “cognitive map” for the operator to identify the Ultrasound Flexion Point (UFP) prior to application. The operator proceeds with the insertion of the vacuum cup while maintaining a mental and visual reference to this frozen sonographic map.

Alignment: The center of the cup must be directed toward the identified UFP. Specifically, the cup’s notch (or indicator line) must be aligned with the sonographically determined Midline Angle to ensure it is positioned along the same axis as the sagittal suture. Since the instrument is not sonographically visible, the operator confirms this alignment either through direct visualization of the notch on the fetal scalp (e.g., by gently parting the maternal labia if the head is not yet crowning) or by tactile feedback, feeling the notch with the fingertip.

Clinical Biofeedback During Traction: During the “pull,” the notch serves as a dynamic clinical indicator. In an occiput anterior (OA) position, the notch should remain stable. In occiput transverse or posterior (OT/OP) positions, as the fetal head undergoes physiological internal rotation, the visually or tactilely monitored notch should rotate synchronously with the descending head. If the operator observes or feels a sudden deviation of the notch independent of fetal head rotation, it may suggest cup slippage (loss of vacuum integrity) rather than physiological internal rotation, prompting the operator to halt traction.

Re-verification: If cup detachment occurs, a rapid translabial scan is repeated to re-map the midline topography before any re-application, significantly reducing the risk of trauma associated with repeated blind attempts [15].

It should be noted that not all vacuum cup models feature a dedicated orientation notch (e.g., Malmström metal cups). In such cases, the traction handle or chain direction serves as a proxy orientation reference; the operator must adapt the biofeedback principle to the specific instrument’s design while maintaining the same goal of aligning the cup center with the sonographically defined midline.

**Figure 1:**
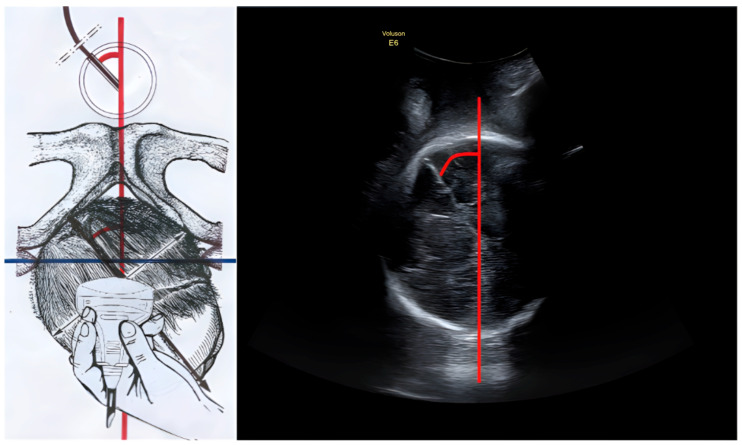
Intrapartum Ultrasound Flexion Point: Correct Application. (**Left**) Schematic representation of a translabial ultrasound scan in the transverse plane (parallel to the interspinous line/bi-ischiatic line, represented as the transverse blue line) of a fetal head in Right Occiput Anterior (ROA) position. The Midline Angle (MLA) is measured (red arc), which is the angle between the infrapubic line (red vertical line) and the fetal midline (black line). The “Ultrasound Flexion Point” is identified as the intersection of the fetal midline (sagittal suture, black line) and the Biparietal Diameter (BPD, black arrow). In the upper panel, the vacuum extractor cup (Kiwi OmniCup) is shown correctly applied along the sagittal suture, centering on the flexion point. (**Right**) The corresponding ultrasound image demonstrating the sonographic midline and the correct alignment.

**Figure 2:**
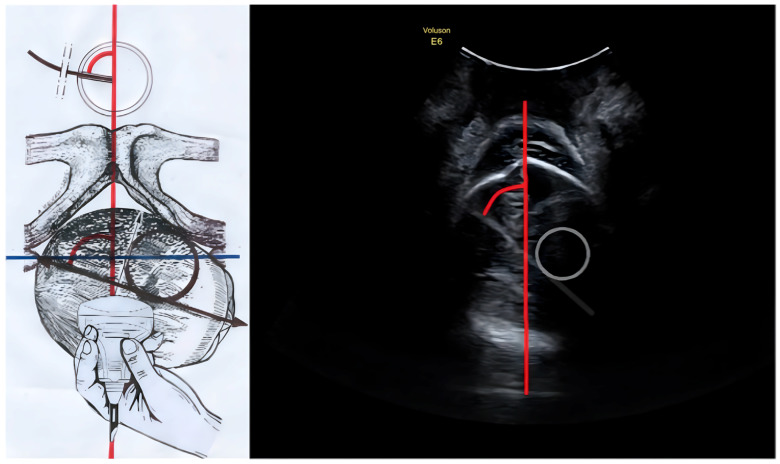
Intrapartum Ultrasound Flexion Point: Incorrect Application. (**Left**) Translabial ultrasound scan in the transverse plane (parallel to the interspinous line/bi-ischiatic line, shown as the transverse blue line) of a fetal head in Right Occiput Anterior (ROA) position, with malrotation as indicated by the MLA (the red arc representing the angle between the red infrapubic line and the fetal midline). It shows the vacuum cup applied incorrectly, with the black outline indicating the cup placed over the left parietal bone and anterior fontanelle, away from the optimal flexion point (midline/BPD intersection). (**Right**) The corresponding ultrasound image highlights the “Incorrect Application Zone” (grey circle) on a fetal head affected by caput succedaneum and moulding, illustrating how the cup deviates from the true sonographic midline (not an actual ultrasound scan since the vacuum cup is not clearly visible on ultrasound).

To illustrate the method, we provide the following representations comparing the schematic anatomical landmarks with the corresponding ultrasound images (Figure 1 and Figure 2).

Furthermore, to address the dynamic nature of this geometric assessment, a step-by-step animation detailing the exact identification of the Ultrasound Flexion Point (UFP) target is provided as Supplementary Video S1.

## 3. Discussion

### 3.1. Instrument Selection and Device Considerations

Instrument choice should be individualized to the clinical scenario and operator competence, as high-quality syntheses consistently show trade-offs between instruments and cup designs. In randomized evidence summarized by Cochrane reviews, forceps are more likely to achieve vaginal birth compared to vacuum extraction but are associated with higher rates of severe maternal perineal trauma [2]. Conversely, while vacuum extraction reduces maternal trauma, it is associated with higher failure rates and an increased risk of neonatal scalp injury [2,39]. Consequently, the “best” instrument is the one most likely to succeed safely given the specific head position, station, urgency, and operator skill set.

When vacuum extraction is selected, cup design significantly influences performance. Historical randomized trials indicate that soft cups are associated with higher failure and detachment rates compared to rigid or metal cups, although they result in fewer scalp injuries [2,39]. This trade-off between convenience and fixation security persists in modern devices. While the Kiwi OmniCup is favored for its versatility, Groom et al. reported that it was associated with a significantly higher failure rate compared to conventional vacuum cups (30.1% vs. 19.2%; RR 1.58; 95% CI 1.10–2.24) and a higher mean number of cup detachments per procedure (0.68 vs. 0.28; *p* < 0.0001) [8]. Similarly, Equy et al. observed that disposable cups exhibited higher detachment rates compared to metallic cups, although they were associated with fewer perineal injuries [7]. Despite these differences in efficacy, randomized and pragmatic trials suggest that severe neonatal outcomes remain broadly comparable between disposable and metal devices [7,8,40]. Furthermore, recent observational evidence highlights an underappreciated “hardware” determinant: larger rigid cups (e.g., 60-mm Malmstrom) may reduce detachment rates and neonatal trauma without increasing maternal morbidity, suggesting cup size should be considered alongside station and rotation needs [41].

### 3.2. Clinical Implications and Risk Factors for Vacuum Failure

Despite the widespread adoption of the vacuum extractor, successful outcomes remain heavily dependent on both operator experience and patient characteristics. Providing a broad perspective on incidence, Panelli et al. analyzed 47,973 operative vaginal delivery attempts, noting that vacuum extraction was the predominant method (93.2%) with a high success rate of 97.3% (compared to 82.4% for forceps) [42]. However, despite this high overall success rate, failures remain a critical concern. Panelli et al. demonstrated that vacuum failure was significantly associated with lower physician procedure volume, as well as clinical factors such as higher maternal BMI and fetal macrosomia (>4000 g) [42].

These findings are echoed by Kane et al., who reported an overall operative vaginal delivery failure rate of 4.7% in a tertiary setting; notably, the majority of these failures (60.7%) involved vacuum extraction and were similarly linked to higher mean birth weights, as well as induction of labor [43]. The variability in clinical performance is further illustrated by Opoku, who reported a failure rate of 6.4% in a teaching hospital setting, underscoring that while vacuum extraction is generally safe, failure rates fluctuate significantly across different practice environments [44]. Collectively, these data suggest that failure is rarely a monovariable event but rather the result of an interaction between device selection, fetal size, and operator volume.

### 3.3. Determinants of Failure and Risk Stratification

While aggregate incidence rates provide a baseline, clinical safety depends on identifying specific predictors of failure and detachment.

#### 3.3.1. Maternal Anthropometry and Analgesia

Nulliparity and maternal stature are foundational risk factors. In a case–control study by Verhoeven et al., where failed vacuum-assisted vaginal delivery (VAVD) occurred in 4.6% of cases, shorter maternal height and nulliparity were identified as independent predictors [45]. Beyond stature, body mass index (BMI) exerts a profound influence. Grasch et al., focusing on nulliparous individuals, demonstrated that a maternal BMI ≥ 30 kg/m^2^ was associated with a failure rate of 8.0%, more than double the 3.4% rate observed in women with a lower BMI [46]. This association is reinforced by a study published in 2023 that emphasized that obesity increases the complexity of operative delivery, heightening risks for both failure and shoulder dystocia [47]. Regarding pain management, contemporary data challenge historical assumptions that neuraxial analgesia predisposes to failure. In a large retrospective cohort study of 7042 primiparous women, Lang Ben Nun et al. found that epidural analgesia (EA) was associated with a significantly lower failure rate compared to women without EA (2.5% vs. 4.2%) [48]. Multivariable analysis confirmed this protective effect (aOR 0.50, 95% CI 0.29–0.85, *p* = 0.01), suggesting that effective pain relief may facilitate pelvic floor relaxation and maternal cooperation, making it a safe option during vacuum trials [48,49].

#### 3.3.2. Fetal Biometrics: Weight Versus Head Circumference

Fetal macrosomia is a well-established predictor of difficulty. Sheiner et al. reported that failed extractions (5.4% incidence) were significantly linked to birth weights >4000 g [50], a threshold also identified by Panelli et al. [42]. However, more granular biometric analysis suggests that head size may be more predictive than absolute weight. Kabiri et al. found that a large head circumference (≥90th percentile) significantly increased the risk of failure with an adjusted odds ratio (aOR) of 2.31 (95% CI 1.70–3.15) [51]. Their analysis indicated that high head circumference was a more specific predictor of cephalopelvic disproportion at the midpelvis than birth weight alone. Conversely, Verhoeven et al. maintained a threshold of ≥3750 g in their prediction model, highlighting the continued relevance of estimated fetal weight in clinical algorithms [45].

#### 3.3.3. Malposition and Determinants of Cup Detachment

Fetal malposition represents the most significant technical challenge to vacuum success. A retrospective case–control study was undertaken at Aga Khan University Hospital, Nairobi, Kenya, by review of medical charts from the period of January 2007 to December 2010, and fetal malposition was identified as the strongest predictor of failure, with a striking Odds Ratio of 12.7 compared to occiput anterior positions [52]. This is critical because malposition is a direct precursor to cup detachment. Schreiber et al., in a retrospective cohort study focused specifically on the mechanism of “pop-offs,” provided granular data on risk factors for detachment [15]. They found that the detachment group was characterized by a high prevalence of occiput posterior (OP) position (70.8% of cases), significantly higher birth weights (>3700 g), and longer durations of vacuum application. These factors—malposition, macrosomia, and prolonged duration—create a “perfect storm” for mechanical failure. Similarly, Le Brun et al. identified deflexed attitude and posterior position as key drivers of failure, which were subsequently linked to lower Apgar scores and craniofacial injuries [53]. The association between malposition and failure is often mediated by incorrect cup placement; audit data from Sau et al. [54] and Cotzias et al. [55] revealed that inaccurate placement occurred in up to 40% of failed cases, underscoring the need for precise identification of the flexion point.

#### 3.3.4. Predictive Utility of Clinical Models

Given these multifactorial risks, the accuracy of clinical prediction remains variable. Palatnik et al., in a case–control study of 4352 women, found that standard clinical factors (race, parity, induction, chorioamnionitis) had only modest predictive capacity, yielding an Area Under the Curve (AUC) of 0.74 [56]. This suggests that clinical judgment alone often fails to capture the geometric complexity of the delivery. In contrast, models that incorporate specific biometric and sonographic data perform significantly better. Verhoeven et al. achieved an AUC of 0.83 using a model combining parity, weight, and station [45]. Even higher accuracy was reported by Rizzo et al., who developed a multiparametric model combining antepartum and intrapartum ultrasound data; their prospective cohort study demonstrated an AUC of 0.913, indicating that integrating objective ultrasound measurements with clinical variables offers the most robust method for predicting vacuum failure [57].

As we have discussed, failure is rarely a single-variable event but the result of complex interactions between maternal anthropometry, fetal biometrics, and dynamic labour factors, and it is further detailed in Table 4.

### 3.4. Biomechanical Principles and Cup Placement Technique

The fundamental biomechanical objective of vacuum extraction is to promote flexion of the fetal head, thereby presenting the smaller suboccipitobregmatic diameter to the birth canal, facilitating descent along the pelvic curve [58,59]. This functional requirement relies entirely on correct cup placement over the “flexion point,” defined as the site along the sagittal suture approximately 3 cm anterior to the posterior fontanelle. Application at this specific landmark is critical not only for efficacy but for minimizing the traction force required.

Achieving this optimal placement, however, presents significant technical challenges, particularly in malpositions. In a simulation study comparing placement techniques, Cuerva et al. demonstrated that the “Vacca technique” (placing the cup while the operator’s finger monitors the flexion point) resulted in significantly higher precision compared to the “Bird technique” [60]. Specifically, the Vacca technique reduced the distance between the cup center and the target landmark, proving superior in transverse and posterior positions where “blind” application is most prone to error.

Deviations from this optimal point have quantifiable mechanical consequences. When the cup is placed off-midline or anteriorly, traction does not align with the pelvic axis, necessitating excessive force to achieve descent. Pettersson et al. quantified this risk, noting that traction forces frequently exceeded the recommended safety threshold of 216 N in 34% of extractions, a variable often underestimated by operators but strongly correlated with detachment and trauma [61]. Real-world audit data confirm that placement error is a dominant cause of procedural failure. Sau et al., evaluating routine practice in the UK, found that incorrect cup placement was evident in 40% of failed vacuum extractions, highlighting a significant training gap in assessing fetal skull orientation [54]. Similarly, Cotzias et al. reported a failure rate of 18.3% in their cohort, with inaccurate placement observed in 21.7% of cases [55]; notably, failure rates were higher in the inaccurate placement group (23% vs. 17%), reinforcing that geometric misalignment is a primary, modifiable determinant of failure.

### 3.5. Pathophysiology of Asynclitism and Head Deflexion

A critical, often underappreciated determinant of vacuum failure and fetal trauma is asynclitism—the lateral deflection of the fetal head. While often considered merely a positional variant, Malvasi et al. (2015) characterize asynclitism as a distinct clinical condition that significantly alters the mechanics of descent [62]. From a biomechanical perspective, Vlasyuk and Malvasi explain that asynclitism leads to an uneven distribution of tension forces on the intracranial structures, specifically the tentorium cerebelli (TC) [13]. Even moderate asynclitism during extraction is considered pathological; if the vacuum cup is applied in a paramedian position (iatrogenic asynclitism), traction exacerbates this lateral tilt, potentially causing one-sided rupture of the TC and subsequent hemorrhage.

This mechanical disadvantage is closely linked to head deflexion. To quantify this, Ghi et al. introduced the “occiput-spine angle” as a novel sonographic parameter. In their study, a narrower occiput-spine angle (<125°)—indicating a deflexed head—was significantly associated with a higher risk of operative delivery and a longer duration of the second stage, whereas an angle >125° favored spontaneous progression [63]. This suggests that the degree of flexion is measurable and directly predictive of mechanical difficulty.

The physical manifestation of these malpositions is fetal head molding. Iversen et al. utilized translabial ultrasound to categorize molding patterns in nulliparous women with slow progress [64,65]. They observed that while occipitoparietal molding was common even in occiput anterior positions, severe molding often reflects the obstruction caused by malposition. When vacuum extraction is attempted on a significantly molded or asynclitic head, the “chignon” formed by the cup may obscure the true bony landmarks, leading to the “blind” misplacement described in the previous section and increasing the risk of failure. This biomechanical environment explains why alternatives like Kielland’s forceps are sometimes considered for transverse arrest [66], though vacuum remains the primary instrument in many settings despite these limitations.

### 3.6. Maternal and Neonatal Outcomes Following Failed Extraction

The clinical decision to attempt vacuum extraction carries distinct risk profiles for the mother and infant, which diverge sharply when the procedure fails. While the Cochrane review by Johanson established that successful vacuum extraction generally reduces maternal perineal trauma compared to forceps, it increases the risk of neonatal cephalohematoma and retinal hemorrhage [39]. However, in the event of failure, the “safety profile” shifts dramatically.

#### 3.6.1. Maternal Morbidity and the Cost of Failure

Failed OVD is a major driver of maternal complications. Kane et al. reported that women who experienced an unsuccessful OVD had a significantly higher rate of postpartum hemorrhage (PPH) compared to those with successful extractions (64.2% vs. 31.5%; *p* < 0.01) [43]. This burden of morbidity is further elucidated by Muraca et al., who analyzed outcomes at midpelvic station [67]; they found that attempted OVD was associated with higher rates of severe obstetric trauma compared to primary cesarean delivery, although severe maternal morbidity (SMM) rates were comparable. When vacuum fails, the management strategy—sequential instrument use versus cesarean section—has a significant impact on maternal outcomes. Melamed et al. [68] and Chamagne et al. [69] both demonstrated that using forceps after a failed vacuum (“sequential instrumentation”) significantly increases the risk of high-grade perineal tears and episiotomy extension compared to proceeding directly to cesarean section. Conversely, Bhide et al. [70] and Miot et al. [71] noted that while cesarean after failed vacuum avoids perineal trauma, it is associated with increased blood loss and longer hospital stays.

#### 3.6.2. Neonatal Morbidity

The fetus bears the primary brunt of a failed extraction. Attali et al. compared cesarean sections performed after failed vacuum attempts against those performed for second-stage arrest without instrumentation [72]. They found that the failed vacuum group had significantly worse neonatal outcomes, including lower umbilical artery pH, higher NICU admission rates, and increased incidence of epicranial hemorrhage. This suggests that the failed attempt itself acts as a “first hit” of trauma before the surgical delivery. This is corroborated by Ahlberg et al., who identified that infants delivered after failed vacuum extraction had higher risks of subgaleal hemorrhage, convulsions, and low Apgar scores compared to successful extractions [73]. Hendler et al. went further, comparing “bad, worse, and worst” scenarios, concluding that vacuum extraction (especially when failed) was associated with higher adverse neonatal outcomes compared to forceps or cesarean delivery [74]. Importantly, baseline neonatal risk in the setting of fetal compromise may be increased by factors independent of the instrument itself, such as nuchal cord, which has been associated with altered acid–base status and variable fetal heart rate patterns [75]. These confounders should be considered when attributing adverse outcomes to the operative procedure per se.

However, data on sequential use are mixed. While Edgar et al. suggested that low forceps delivery following failed Kiwi OmniCup extraction had minimal neonatal morbidity (cephalohematoma 19.8%, no intracranial hemorrhage) [76], Al-Kadri et al. [77] and De Jonge et al. [78] warned that sequential use increased neonatal morbidity and was linked to neonatal deaths in cases of multiple instrumentation.

### 3.7. Intrapartum Ultrasound: Diagnostic Accuracy, Evidence, and Guidelines

The reliance on DE to identify the flexion point is problematic, particularly in difficult labors where caput succedaneum masks the sutures. Akmal et al. demonstrated that DE fails to identify the correct position in approximately 25% of cases overall, with accuracy dropping to 54% for occiput-lateral and posterior positions [11]. ITU offers superior diagnostic precision. Wong et al. provided quantitative evidence of this benefit in a controlled study, showing that the mean distance between the cup center and the optimal flexion point was significantly smaller in the ultrasound-assisted group compared to DE (2.1 ± 1.3 cm vs. 2.8 ± 1.0 cm, *p* = 0.039) [79]. Furthermore, Garcia-Jimenez et al. found that the concordance rate between DE and ITU was only 46.1% for occiput posterior positions, establishing ultrasound as the reference standard in complex cases [80].

Specialty guidance increasingly positions ITU as decision support. International Society of Ultrasound in Obstetrics and Gynecology (ISUOG) guidelines emphasize standardized approaches for transabdominal and translabial assessment but position ultrasound as an adjunct rather than a mandatory standard of care [81]. Conversely, the Central European DGGG/ÖGGG/SGGG guideline explicitly recommends ultrasound prior to vaginal-operative birth when palpation does not allow accurate assessment of head position and rotation [32]. However, improved diagnosis does not automatically translate to better outcomes. In the multicenter R.I.S.P.O.S.T.A. randomized trial, while adding transabdominal sonography reduced misclassification of fetal head position, the trial was stopped early for futility and did not demonstrate reductions in failed vacuum extraction or maternal/neonatal morbidity [82]. Similarly, systematic review by Mappa et al. [26] found no reduction in cesarean rates compared to routine care, though they supported improved diagnostic certainty and standardization of decision-making.

Beyond position, ITU-derived measures provide prognostic information.

- Head–Perineum Distance (HPD): Kahrs et al. [22] established that an HPD ≤ 25 mm is associated with shorter extraction duration (median 6.0 vs. 8.0 min, *p* < 0.01) and lower cesarean conversion rates (3.9% vs. 22.0%) compared to HPD > 35 mm.
- Angle of Progression (AoP): Sainz et al. [20] identified AoP < 105° and Midline Angle > 45° as “very unfavorable” parameters. Expanding on this, a meta-analysis by Nassr et al. [24] confirmed that AoP measured during the second stage predicts uncomplicated operative vaginal delivery with high sensitivity and specificity, particularly in nulliparous women.
- Comparative Accuracy: A broader comparative meta-analysis by Skinner et al. [25] reported high prognostic accuracy for AoP and progression distance, moderate accuracy for head direction and HPD, and fair accuracy for midline angle, suggesting that multiple measures have clinically useful prognostic value depending on the context.

### 3.8. Standardized Pre-Application Workflow and Technical Pitfalls

From an ultrasound-oriented perspective, adopting a standardized workflow improves the reliability of intrapartum findings and their translation into operative planning. While ISUOG guidelines emphasize that intrapartum ultrasound complements rather than replaces clinical assessment [81], a pragmatic pre-application protocol is essential for safety. Adapted from ISUOG recommendations and common protocols, the workflow includes:

- Transabdominal Ultrasound: Confirm fetal head position (occiput orientation) and, crucially, exclude face or brow presentations which would contraindicate vacuum use.
- Translabial Ultrasound (Sagittal Plane): Assess fetal descent and station using reproducible parameters such as the Angle of Progression (AoP) and/or Head–Symphysis Distance (HSD)—our preferred metric for the reasons detailed in Step 2—or Head–Perineum Distance (HPD), recognizing local nomenclature and device-specific measurement conventions. As noted by Nassr et al. [24], AoP is a validated predictor of uncomplicated delivery and should inform the need for senior support or a “trial” in theatre for higher-risk cases.
- Translabial Ultrasound (Transverse Plane): Assess the midline (sagittal suture) orientation and head rotation. Document any marked asynclitism, molding, or caput succedaneum that may limit the accuracy of subsequent digital examination.
- Clinical Integration: Synthesize ultrasound findings with standard guideline prerequisites—full dilatation, ruptured membranes, engaged head, adequate analgesia, empty bladder, and availability of escalation—to decide whether to proceed with vacuum, consider rotational forceps, or escalate to second-stage cesarean depending on urgency and operator competence.

The implementation of this workflow requires awareness of specific technical limitations. Key pitfalls include the difficulty of obtaining a true mid-sagittal plane in the presence of advanced molding or caput, and the potential misinterpretation of transverse planes when the head is markedly asynclitic. Furthermore, acoustic windows may be reduced in maternal obesity or when the perineum is crowded by active pushing. To mitigate these risks, units implementing intrapartum ultrasound in operative birth should establish minimum training standards, image archiving for audit, and interobserver calibration [81,83,84].

## 4. Technical Proposal: The Ultrasound Flexion Point (UFP) Method

Despite the clear diagnostic advantages of intrapartum ultrasound, systematic reviews noted that sonographic knowledge of fetal position alone did not significantly reduce the incidence of failed operative deliveries in general practice [26,27]. We hypothesize this is because ITU is typically used before the procedure, but not during the critical moment of cup application, leaving the operator to navigate the “blind zone” of the caput succedaneum manually. To bridge this gap, we propose the Ultrasound Flexion Point (UFP) method. This approach shifts the paradigm from “static diagnosis” to “non-real-time orientation feedback”.

### 4.1. Definition and Rationale

In routine clinical settings, the vacuum cup is not consistently visualized during translabial ultrasound-guided application; therefore, the realistic value of ultrasound is to provide an accurate pre-application “map”.

- The UFP is identified on a frozen transverse translabial ultrasound image as the intersection between (i) the fetal cranial midline (sonographic proxy of the sagittal suture) and (ii) the biparietal diameter (BPD) plane on the same transverse view.
- It is not claimed to be anatomically identical to the classical flexion point. Rather, it is a pragmatic sonographic orientation landmark intended to approximate the target zone and reduce spatial uncertainty, particularly when caput succedaneum and molding make digital palpation of fontanelles unreliable (i.e., when fontanelles become palpable “ghosts”). Geometrically, in a well-flexed head with minimal molding, the BPD plane crosses the sagittal suture in the parietal region, typically within 1–2 cm of the posterior fontanelle—close to the classical flexion point (~3 cm anterior to it). As molding elongates the skull or caput displaces the soft-tissue contour, the true bony BPD–midline intersection may shift slightly cephalad; the operator should therefore interpret the UFP as the centre of a target zone rather than a fixed point, and re-scan after any detachment to account for progressive deformation. We acknowledge that in cases of severe cranial molding (e.g., dolichocephaly), the plastic elongation of the fetal skull may cause a slight axial translation of the BPD plane relative to the anatomical flexion point. However, for the practical purpose of positioning a standard 6-cm vacuum cup, this sonographic intersection remains a clinically reliable spatial proxy that is significantly superior to blind palpation through a massive caput succedaneum, as supported by the molding pattern data reported by Iversen et al. [64,65].

The anatomical basis for this approximation rests on the ovoid morphology of the fetal calvarium: the occipital pole is broader than the frontal pole, so that the parietal eminences—which define the BPD—are displaced posteriorly relative to the longitudinal midpoint of the skull. In a well-flexed term fetus, the suboccipitobregmatic diameter (9.5 cm) equals the BPD (9.5 cm). The classical flexion point lies on the sagittal suture ~3 cm anterior to the lambda and ~6 cm posterior to the bregma [9,85]; the sagittal suture therefore spans approximately 9 cm from bregma to lambda (i.e., 6 + 3 cm), placing the flexion point in the posterior third of the sagittal axis—precisely where the transverse BPD plane intersects the midline. On translabial ultrasound, the BPD plane is identified as the level of maximum biparietal width with symmetric visualization of the thalami; its intersection with the echogenic midline therefore falls within 1–2 cm of the classical flexion point in the absence of severe molding. This geometric convergence provides the anatomical rationale for using the UFP as a pragmatic sonographic proxy. When a standard 6-cm vacuum cup is centred on this intersection, its posterior rim approaches the lambda while its anterior rim remains ~3 cm from the bregma, replicating the geometry described for optimal cup placement in the “Vacca 5-Steps” technique [9,60].

Although the cup itself is typically not visible on ultrasound, the frozen transverse image establishes a consistent spatial map. During the subsequent digital application, this map serves as a “cognitive anchor”: the operator aims to keep the cup center aligned with the sagittal suture and to position the center as close as feasible to the UFP-based target. This provides “non-real-time orientation feedback.” Unlike fontanelles, which become palpable “ghosts” under severe molding, the BPD-Midline intersection remains sonographically distinct. This ensures that the cup is centered on the sagittal suture, avoiding paramedian application. Step 4 of the bedside method and Step 5 of the full algorithm (Section 4.2) both rely on the alignment of the vacuum cup’s indicator notch with the sonographically defined midline. If the notch deviates during traction, this may suggest incomplete rotation, progressive asynclitism, or cup slippage. This allows the operator to halt and readjust before a traumatic detachment occurs, directly countering the risk factors of multiple pulls identified by [86] and targeting the prevention of pathological asynclitism [13].

### 4.2. Algorithm: Pre-Application Intrapartum Ultrasound and UFP-Based Orientation Workflow

**Figure 3:**
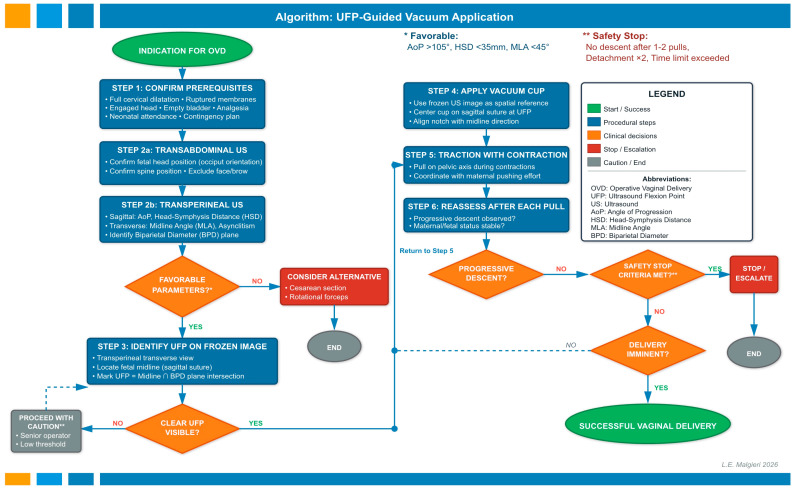
Flowchart for Ultrasound Flexion Point (UFP)-Guided Vacuum Application.

We propose a standardized seven-step algorithm for UFP-based vacuum application, integrating prerequisite verification, diagnostic ultrasound assessment, UFP identification, cup application with visual biofeedback, and continuous safety reassessment (Figure 3). The algorithm is structured around five critical decision points that mandate escalation when objective safety criteria are violated.

This flowchart illustrates the standardized 7—step algorithm for UFP-based vacuum-assisted delivery with integrated decision points and safety checkpoints. The workflow begins with confirmation of indication for operative vaginal delivery (OVD) and proceeds through prerequisite verification, diagnostic ultrasound assessment, UFP identification, cup application with spatial orientation guidance, traction technique, continuous reassessment, and delivery completion. Color coding indicates element function: green (start/successful outcome), blue (procedural steps), orange (clinical decision points), red (stop/escalation), and gray (caution/alternative pathways). Dashed arrows represent iterative loops when safety criteria are not yet met but progressive descent continues. Key decision points include: (1) Assessment of favorable ultrasound parameters (AoP > 105°, HSD < 35 mm, MLA < 45°, minimal asynclitism) to proceed versus consider alternative delivery mode; (2) Verification of clear UFP visibility on frozen translabial image; (3) Evaluation of progressive descent after each pull; (4) Application of safety stop criteria (no descent after 1–2 pulls, detachment ×2, time limit exceeded); and (5) Assessment of delivery imminence. The algorithm emphasizes that when clear UFP visualization is not achievable, operators should proceed with enhanced caution using digital examination, senior supervision, and a low threshold for discontinuation. The return loop from Decision 5 (delivery not imminent) to Step 5 (traction) allows for continued attempts when progressive descent is demonstrated and safety criteria are not violated, while the stop pathway from Decision 4 mandates immediate escalation to cesarean section or alternative instrumentation when safety thresholds are exceeded.

The workflow begins with confirmation of standard prerequisites (STEP 1) followed by comprehensive ultrasound evaluation (STEP 2a–b): transabdominal assessment to confirm head position and exclude malpresentations, and translabial assessment in sagittal and transverse planes to measure prognostic parameters (AoP, HSD, MLA, asynclitism degree). The first decision checkpoint (DECISION 1) evaluates whether ultrasound parameters are favorable for proceeding; unfavorable parameters (AoP < 105°, HSD > 35 mm, MLA > 45°) trigger consideration of alternative delivery modes.

The core innovation involves identification of the Ultrasound Flexion Point (UFP) on the frozen translabial transverse image (STEP 3), defined as the intersection of the fetal midline and the biparietal diameter plane. This sonographic landmark serves as a spatial reference during digital-guided cup application (STEP 4), with the cup’s notch aligned to the midline for subsequent spatial orientation guidance. If UFP visualization is suboptimal due to severe caput or poor acoustic window (DECISION 2), the operator may return iteratively to STEP 3 or proceed with enhanced caution using standard digital examination with senior supervision.

Traction is applied during contractions along the pelvic axis (STEP 5), with systematic reassessment after each pull (STEP 6) evaluating progressive descent, cup position, and maternal–fetal status. Three sequential decision checkpoints govern continuation versus escalation: DECISION 3 confirms progressive descent, DECISION 4 applies mandatory safety stop criteria (no descent after 1–2 pulls, detachment ×2, time limit exceeded), and DECISION 5 assesses delivery imminence. When delivery is not imminent, but descent continues within safety parameters, the algorithm returns iteratively to STEP 5. When safety criteria are violated, immediate escalation to cesarean section or alternative instrumentation is mandated.

This decision-driven framework prioritizes timely abandonment over persistent extraction, reflecting evidence that protracted attempts (>15 min, >6 pulls, >2 detachments) increase neonatal intracranial hemorrhage risk nine-fold [86]. The UFP method adds approximately 2–3 min to pre-application assessment. Implementation requires operator competency in translabial ultrasound, definition of local parameter thresholds, and quality assurance through post-delivery chignon-to-target distance audits. Complete algorithmic details, decision criteria, color-coded pathways, and abbreviations are provided in Figure 3 and its legend.

### 4.3. Advantages and Limitations

The UFP method offers several potential advantages over traditional digital examination-guided cup placement. First, it provides an objective sonographic proxy that remains sonographically identifiable even when caput succedaneum obscures the fontanelles and sutures that are traditionally used for tactile orientation. This is particularly valuable in prolonged labor with significant molding, precisely the clinical scenario where correct cup placement is most challenging yet most critical for success. Second, the method may be especially beneficial in occipitoposterior and occipitotransverse positions, where digital examination accuracy is known to be lowest [11] and where the risk of paramedian application is highest. Third, the UFP approach provides a standardized training framework for less-experienced operators, translating abstract ultrasound diagnosis into concrete spatial targets for cup application. This may accelerate the learning curve and improve consistency across different operator skill levels. Fourth, the non-real-time biofeedback provided by observing the cup notch alignment during traction offers a mechanism to detect early cup slippage or incomplete rotation, potentially allowing the operator to halt and readjust before traumatic detachment occurs. Finally, by potentially reducing cup detachment rates and improving first-attempt success, the method may contribute to reductions in neonatal trauma, including subgaleal hematoma and intracranial hemorrhage.

However, several important limitations must be acknowledged. First and most critically, the UFP is a cognitive aid, not real-time ultrasound-guided cup placement. The vacuum cup itself is typically not visible on translabial ultrasound during application, and the method relies on the operator’s ability to translate a frozen pre-application image into accurate digital placement. Second, the technique requires proficiency in intrapartum translabial ultrasound, including the ability to obtain adequate transverse views and accurately identify the fetal midline and biparietal diameter. This necessitates dedicated training and may not be immediately available in all practice settings. Third, the UFP method adds time to the procedure, estimated at 2–3 min for the additional ultrasound assessment. While this is unlikely to be clinically significant in non-urgent situations, it may limit applicability in Category 1 emergencies requiring immediate delivery. Fourth, acoustic window limitations may preclude adequate visualization in some cases, particularly in women with high body mass index or in the presence of very severe caput succedaneum that distorts the anatomical landmarks even on ultrasound. Fifth, the method has not yet been validated in prospective trials; all potential benefits remain theoretical pending rigorous evaluation in controlled studies. The technique should therefore be considered experimental and implemented only within research protocols or quality improvement initiatives with appropriate oversight.

Units adopting this approach must establish standardized training programs to ensure operator competence in both intrapartum ultrasound acquisition and UFP identification. Quality assurance metrics should be defined, including inter-operator agreement on UFP location (κ coefficient) and post-delivery audit of chignon-to-target distance. Local protocols must be adapted to specify when UFP assessment is indicated (e.g., prolonged second stage with caput, suspected malposition) versus when it may be omitted (e.g., straightforward occiput anterior position with clear landmarks on digital examination). Most importantly, implementation must occur within a safety culture that prioritizes strict adherence to established stop rules for operative vaginal delivery, as the UFP method is intended to improve technique, not to encourage attempts in unsuitable cases.

Regarding the concern that intrapartum ultrasound may be impractical during the time-pressured moment of vacuum cup insertion, it is important to distinguish between two phases with different ultrasound requirements. The comprehensive four-parameter assessment (AoP, HSD, MLA, AD) is performed before the decision to proceed with vacuum extraction. This pre-procedural evaluation—which requires approximately 2–3 min—informs case selection, feasibility, and risk stratification, and is performed when the clinical team is still evaluating the mode of delivery. Once the decision to apply the vacuum has been made, the UFP-specific ultrasound step is considerably simpler and faster: it requires only a single frozen transverse translabial image to identify the midline and the BPD plane—a task that typically takes less than 30 s for an operator familiar with intrapartum ultrasound. The probe is then removed, and the frozen image serves as the cognitive map for cup placement. Crucially, the operator who places the vacuum cup need not be the same person who performs the ultrasound scan. In a team-based delivery room setting, one member of the team (e.g., a sonographer, a midwife trained in intrapartum ultrasound, or a second obstetrician) can acquire and freeze the transverse image while the primary operator prepares for cup insertion. This division of tasks mirrors the teamwork model already standard in operative vaginal delivery, where one clinician manages the instrument while others monitor fetal heart rate, manage analgesia, or assist with maternal positioning. If cup detachment occurs, a brief re-scan (again < 30 s) to re-identify the midline orientation is recommended before re-application, but no repeat measurement of AoP, HSD, or AD is needed at that stage. A recognized limitation of this method is its reliance on the operator’s spatial proprioception to translate a two-dimensional pre-application sonographic map into physical alignment during blind digital insertion. Future validation studies should incorporate pelvic trainers and simulation models to quantify the accuracy of this cognitive mapping and establish a specific learning curve for novice operators.

The proposal of the Ultrasound Flexion Point (UFP) warrants rigorous validation before broad clinical implementation. Future research efforts should be structured around three key domains:

1. Feasibility and Reliability: Prospective evaluations must prioritize technical feasibility metrics, specifically the time added to the procedure and the proportion of cases where a clear UFP can be obtained. Reliability studies should assess intra- and inter-operator agreement on UFP identification and on the associated midline/rotation measures. Objective placement audits—measuring the post-delivery chignon-to-target distance—will be essential to quantify geometric precision.
2. Clinical Endpoints: Pragmatic trials should focus on safety-critical outcomes. Primary endpoints must include cup detachment rates, the incidence of failed vacuum extraction, and the need for sequential instrumentation. Secondary endpoints should capture decision-to-delivery time and composite maternal and neonatal morbidity.
3. Added Value and Training: Because ultrasound already supports case selection through feasibility metrics (AoP, progression distance, HPD), future studies must test whether adding UFP-based orientation improves outcomes beyond what would be expected from improved selection alone. Furthermore, embedded training evaluations are essential. As noted by Bahl et al. [87] and Ubom et al. [30], skill acquisition and human factors remain primary determinants of operative vaginal birth safety; therefore, research should assess whether UFP-based protocols improve the learning curve for novice operators compared to standard digital training.

As highlighted in recent literature, the clinical utility of intrapartum ultrasound markers depends heavily on measurement reliability. Future UFP studies must follow established repeatability and reproducibility frameworks used for ultrasound-based labor parameters [88]. Practical mastery can be assessed in the delivery center via supervised scans and post-delivery audits measuring the chignon-to-target distance.

Currently, the primary limitation of this proposal is the absence of small-scale in vivo data. The immediate next step is to conduct a pilot feasibility study within our delivery center to provide preliminary data on practical implementation times, operator satisfaction, and early safety metrics before progressing to a larger randomized trial.

## 5. Conclusions

The vacuum extractor remains an indispensable instrument in modern obstetric practice, offering a critical alternative to second-stage cesarean delivery. However, its safety profile is intrinsically linked to the operator’s ability to achieve a biomechanically precise application within strict prerequisites and predefined stop rules. The literature reviewed in this manuscript highlights a persistent “safety gap”: while devices like the Kiwi OmniCup have improved ease of use, they are associated with higher rates of detachment and failure when applied without geometric precision. The consequences of these technical errors are not benign, as demonstrated by the significant association between cup detachments, iatrogenic asynclitism, and severe neonatal morbidity, including subgaleal and intracranial hemorrhage.

Traditional reliance on digital vaginal examination for identifying the flexion point is fraught with inaccuracy, particularly in the very cases where vacuum assistance is most indicated—prolonged labors complicated by caput succedaneum and molding. While intrapartum ultrasound has established itself as the gold standard for diagnosing fetal position and station (offering quantitative prognostic metrics like AoP), its potential to actively navigate the procedure has remained largely untapped. Real-time visualization of the vacuum cup is not routinely achievable, leading to a paradox where improved diagnosis does not consistently translate into better clinical outcomes.

We propose the “Intrapartum Ultrasound Flexion Point” (UFP) method as a standardized clinical protocol to bridge this gap. By utilizing the intersection of the fetal midline and the Biparietal Diameter (BPD) as an objective, sonographically visible target, this method eliminates the subjectivity of “blind” palpation. It offers a pragmatic, non-real-time orientation feedback strategy: the UFP serves as a “cognitive anchor” to improve operator spatial orientation during cup application, while the alignment of the cup’s notch provides a check against slippage. This proposal advocates for a paradigm shift from “static diagnosis” to “pre-procedural spatial orientation guidance” in operative vaginal delivery. We posit that the adoption of this method could significantly reduce the rates of application failure and vacuum-associated fetal trauma. Future research must now focus on validating this technique through prospective, randomized controlled trials to quantify its impact on neonatal safety metrics and extraction success rates in diverse clinical settings.

It is important to emphasize that, while the UFP method is supported by strong biomechanical rationale and addresses documented limitations of current practice, it remains an experimental technique requiring rigorous prospective validation. Until evidence from adequately powered randomized controlled trials confirms its clinical utility, safety profile, and generalizability across different operator experience levels and clinical settings, the UFP method should be implemented only within research protocols or quality improvement initiatives with appropriate institutional oversight and informed consent. The present manuscript serves to introduce the technique, establish its theoretical foundation, and provide a framework for future validation studies.

## Figures and Tables

**Table 2:** Practical checklist before and during vacuum-assisted birth (expert synthesis).

Confirm indication and discuss alternatives; obtain consent proportionate to urgency.
Confirm prerequisites: full cervical dilatation, ruptured membranes, engaged head, known position and station, clinical assessment of pelvic adequacy, empty bladder, adequate analgesia, and immediate access to neonatal support.
If position/station are uncertain on digital examination, perform intrapartum ultrasound (transabdominal and/or translabial) to confirm head position, rotation, and descent.
Choose an instrument within operator competence; plan the fallback pathway (forceps vs. second-stage cesarean) before starting; consider theatre for higher-risk ‘trial’ procedures.
Ensure correct cup placement over the sagittal suture and at/near the intended flexion-point target; exclude maternal tissue.
Apply traction in the pelvic axis during contractions with coordinated maternal effort; reassess after each pull.
Stop and escalate if correct application cannot be achieved easily, if progressive descent is not observed after 1–2 pulls, if detachments occur beyond acceptable thresholds, or if maternal/fetal status deteriorates.
Avoid sequential instruments where possible; if sequential instrumentation is considered, document rationale and alert the neonatal team for enhanced surveillance.

**Table 3:** Documentation checklist for operative vaginal birth, informed by international guideline recommendations [1,28,29,30,35] (expert synthesis).

Indication(s) for operative vaginal birth and urgency category; documented counselling/consent proportional to urgency.
Pre-procedure examination: cervical dilatation, membrane status, fetal head position (digital and/or ultrasound), station/descent (including ultrasound parameters when used), and degree of caput/molding.
Fetal status at decision-to-deliver (cardiotocography [CTG] category)
Analgesia/anesthesia, bladder status (emptied/catheterised), and personnel present (including senior supervision)
Instrument details: device type (vacuum/forceps), cup type/size, suction mode (if relevant), and planned back-up strategy.
Procedure metrics: number of pulls, number of cup detachments/pop-offs, total application time, and reasons for discontinuation/escalation (if applicable).
Maternal outcomes: episiotomy (type), perineal trauma grade, estimated blood loss, and other complications.
Neonatal outcomes: Apgar scores, cord gases if obtained, scalp/facial injuries, cephalohematoma/subgaleal hemorrhage concerns, and neonatal team evaluation/plan.

**Table 4:** Summary of significant risk factors for vacuum extraction failure and adverse outcomes identified in the reviewed literature.

Study	Study Type	Identified Risk Factors/Key Findings	Statistical Association/Outcome
Verhoeven et al. [45]	Case–Control	Nulliparity, Fetal weight ≥ 3750 g, Occiput Posterior (OP), High head station	Prediction model accuracy: AUC 0.83 (95% CI 0.77–0.90)
Schreiber et al. [15]	Retrospective Cohort	Risk factors for Detachment: OP position (70.8%), Birth weight > 3700 g, Longer vacuum duration	Detachment linked to subgaleal hemorrhage (SGH) (*p* = 0.001) and Acidosis (*p* = 0.03)
Lang Ben Nun [48]	Retrospective Cohort	Epidural analgesia reduces failure risk in primiparous women	Failure rate: 2.5% (Epidural) vs. 4.2% (No Epidural); aOR 0.50 (95% CI 0.29–0.85)
Kabiri et al. [51]	Retrospective Analysis	Large Head Circumference (≥90th percentile) is a specific predictor	aOR 2.31 (95% CI 1.70–3.15) for failure associated with large head circumference
Grasch et al. [46]	Retrospective Cohort	Maternal Obesity (BMI ≥ 30 kg/m^2^)	Failure rate: 8.0% (Obese) vs. 3.4% (Non-Obese)
Wanyonyi et al. [52]	Case–Control	Fetal Malposition strongly linked to failure	Malposition OR 12.7 (95% CI 1.5–14.8) for failure
Rizzo et al. [57]	Prospective Cohort	Multiparametric model (Head Circumference, Subpubic Angle, Maternal Height)	High predictive accuracy: AUC 0.913
Sheiner et al. [50]	Retrospective	Birth weight > 4000 g, Lack of prenatal care	Failure rate: 5.4%; linked to higher fetal/maternal complications

## Data Availability

No new data were created or analyzed in this study. Data sharing is not applicable to this article.

## Supplementary Materials

The following supporting information can be downloaded at https://www.mdpi.com/article/10.3390/diagnostics16060946/s1, Video S1: Step-by-step animation of the Ultrasound Flexion Point (UFP) identification, demonstrating probe placement, visualization of the fetal midline, assessment of the Midline Angle (MLA), identification of the Biparietal Diameter (BPD) plane, and the final intersection defining the UFP target.

## Abbreviations

The following abbreviations are used in this manuscript:

AIDA	Artificial Intelligence Dystocia Algorithm
AD	Asynclitism Degree
AoP	Angle of Progression
AUC	Area Under the Curve
aOR	Adjusted Odds Ratio
BPD	Biparietal Diameter
BMI	Body Mass Index
CTG	Cardiotocography
DE	Digital Examination
DGGG/ÖGGG/SGGG	Deutsche/Österreichische/Schweizerische Gesellschaft für Gynäkologie und Geburtshilfe
EA	Epidural Analgesia
HPD	Head–Perineum Distance
HSD	Head–Symphysis Distance
ITU	Intrapartum Ultrasound
MLA	Midline Angle
NICU	Neonatal Intensive Care Unit
OA	Occiput Anterior
OP	Occiput Posterior
OT	Occiput Transverse
OVD	Operative Vaginal Delivery
PPH	Postpartum Hemorrhage
UFP	Ultrasound Flexion Point
ACOG	American College of Obstetricians and Gynecologists
ROA	Right Occiput Anterior
RCOG	Royal College of Obstetricians and Gynaecologists
RR	Relative Risk
FIGO	International Federation of Gynecology and Obstetrics
RANZCOG	Royal Australian and New Zealand College of Obstetricians and Gynaecologists
ISUOG	International Society of Ultrasound in Obstetrics and Gynecology
SGH	Subgaleal Haemorrhage
SMM	Severe Maternal Morbidity
SOGC	Society of Obstetricians and Gynaecologists of Canada
TC	Tentorium Cerebelli
VAVD	Vacuum-Assisted Vaginal Delivery
NICE	National Institute for Health and Care Excellence
FEBRASGO	Brazilian Federation of Gynecology and Obstetrics Associations

## References

[1] American College of Obstetricians and Gynecologists (2020). Operative Vaginal Birth: ACOG Practice Bulletin, Number 219. Obstet. Gynecol..

[2] Verma G.L., Spalding J.J., Wilkinson M.D., Hofmeyr G.J., Vannevel V., O’Mahony F. (2021). Instruments for Assisted Vaginal Birth.

[3] Vacca A. (2001). Operative Vaginal Delivery: Clinical Appraisal of a New Vacuum Extraction Device. Aust. N. Z. J. Obstet. Gynaecol..

[4] Vacca A. (2006). Vacuum-assisted Delivery: An Analysis of Traction Force and Maternal and Neonatal Outcomes. Aust. N. Z. J. Obstet. Gynaecol..

[5] Wang Y., Niu Y., Xu Z., Yan X., Li J., Xu H. (2024). Association of the Kiwi OMNICUP System with Maternal and Neonatal Morbidity: A Retrospective Cohort Study. Int. J. Gynecol. Obstet..

[6] Turkmen S. (2015). Maternal and Neonatal Outcomes in Vacuum-assisted Delivery with the Kiwi OmniCup and Malmström Metal Cup. J. Obstet. Gynaecol. Res..

[7] Equy V., David-Tchouda S., Dreyfus M., Riethmuller D., Vendittelli F., Cabaud V., Langer B., Margier J., Bosson J.-L., Schaal J.-P. (2015). Clinical Impact of the Disposable Ventouse iCup^®^ versus a Metallic Vacuum Cup: A Multicenter Randomized Controlled Trial. BMC Pregnancy Childbirth.

[8] Groom K., Jones B., Miller N., Paterson-Brown S. (2006). A Prospective Randomised Controlled Trial of the Kiwi Omnicup versus Conventional Ventouse Cups for Vacuum-assisted Vaginal Delivery. BJOG Int. J. Obstet. Gynaecol..

[9] Vacca A. (2004). Vacuum-Assisted Delivery: Practical Techniques to Improve Patient Outcomes. OBG Manag..

[10] Parente M.P., Natal Jorge R.M., Mascarenhas T., Fernandes A.A., Silva-Filho A.L. (2010). Computational Modeling Approach to Study the Effects of Fetal Head Flexion during Vaginal Delivery. Am. J. Obstet. Gynecol..

[11] Akmal S., Kametas N., Tsoi E., Hargreaves C., Nicolaides K.H. (2003). Comparison of Transvaginal Digital Examination with Intrapartum Sonography to Determine Fetal Head Position before Instrumental Delivery. Ultrasound Obstet. Gynecol..

[12] Kong C.W., To W.W.K. (2023). Precision of Vacuum Cup Placement and Its Association with Subgaleal Hemorrhage and Associated Morbidity in Term Neonates. Arch. Gynecol. Obstet..

[13] Vlasyuk V., Malvasi A. (2022). The Importance of Asynclitism in Birth Trauma and Intrapartum Sonography. J. Matern. Fetal Neonatal Med..

[14] Caputo F., Caristo I., Barranco R., Vallega Bernucci L., Torielli F., Ventura F. (2025). A Rare Case of Neonatal Death Due to a Cranial Vault Fracture Following the Use of Obstetric Vacuum Extractor and Review of Literature. Am. J. Forensic Med. Pathol..

[15] Schreiber H., Cohen G., Farladansky-Gershnabel S., Shechter Maor G., Sharon-Weiner M., Biron-Shental T. (2022). Adverse Outcomes in Vacuum-Assisted Delivery after Detachment of Non-Metal Cup: A Retrospective Cohort Study. Arch. Gynecol. Obstet..

[16] Chou M.R., Kreiser D., Taslimi M.M., Druzin M.L., El-Sayed Y.Y. (2004). Vaginal versus Ultrasound Examination of Fetal Occiput Position during the Second Stage of Labor. Am. J. Obstet. Gynecol..

[17] Sherer D.M., Miodovnik M., Bradley K.S., Langer O. (2002). Intrapartum Fetal Head Position I: Comparison between Transvaginal Digital Examination and Transabdominal Ultrasound Assessment during the Active Stage of Labor. Ultrasound Obstet. Gynecol..

[18] Ramphul M., Kennelly M., Murphy D.J. (2012). Establishing the Accuracy and Acceptability of Abdominal Ultrasound to Define the Foetal Head Position in the Second Stage of Labour: A Validation Study. Eur. J. Obstet. Gynecol. Reprod. Biol..

[19] Sherer D.M., Miodovnik M., Bradley K.S., Langer O. (2002). Intrapartum Fetal Head Position II: Comparison between Transvaginal Digital Examination and Transabdominal Ultrasound Assessment during the Second Stage of Labor. Ultrasound Obstet. Gynecol..

[20] Sainz J.A., Borrero C., Aquise A., Serrano R., Gutiérrez L., Fernández-Palacín A. (2016). Utility of Intrapartum Transperineal Ultrasound to Predict Cases of Failure in Vacuum Extraction Attempt and Need of Cesarean Section to Complete Delivery. J. Matern. Fetal Neonatal Med..

[21] Bultez T., Quibel T., Bouhanna P., Popowski T., Resche-Rigon M., Rozenberg P. (2016). Angle of Fetal Head Progression Measured Using Transperineal Ultrasound as a Predictive Factor of Vacuum Extraction Failure. Ultrasound Obstet. Gynecol..

[22] Kahrs B.H., Usman S., Ghi T., Youssef A., Torkildsen E.A., Lindtjørn E., Østborg T.B., Benediktsdottir S., Brooks L., Harmsen L. (2017). Sonographic Prediction of Outcome of Vacuum Deliveries: A Multicenter, Prospective Cohort Study. Am. J. Obstet. Gynecol..

[23] Kasbaoui S., Séverac F., Aïssi G., Gaudineau A., Lecointre L., Akladios C., Favre R., Langer B., Sananès N. (2017). Predicting the Difficulty of Operative Vaginal Delivery by Ultrasound Measurement of Fetal Head Station. Am. J. Obstet. Gynecol..

[24] Nassr A.A., Hessami K., Berghella V., Bibbo C., Shamshirsaz A.A., Shirdel Abdolmaleki A., Marsoosi V., Clark S.L., Belfort M.A., Shamshirsaz A.A. (2022). Angle of Progression Measured Using Transperineal Ultrasound for Prediction of Uncomplicated Operative Vaginal Delivery: Systematic Review and Meta-analysis. Ultrasound Obstet. Gynecol..

[25] Skinner S.M., Giles-Clark H.J., Higgins C., Mol B.W., Rolnik D.L. (2023). Prognostic Accuracy of Ultrasound Measures of Fetal Head Descent to Predict Outcome of Operative Vaginal Birth: A Comparative Systematic Review and Meta-Analysis. Am. J. Obstet. Gynecol..

[26] Mappa I., Tartaglia S., Maqina P., Makatsariya A., Ghi T., Rizzo G., D’Antonio F. (2021). Ultrasound vs Routine Care before Instrumental Vaginal Delivery: A Systematic Review and Meta-analysis. Acta Obstet. Gynecol. Scand..

[27] Bellussi F., Di Mascio D., Salsi G., Ghi T., Dall’Asta A., Zullo F., Pilu G., Barros J.G., Ayres-de-Campos D., Berghella V. (2022). Sonographic Knowledge of Occiput Position to Decrease Failed Operative Vaginal Delivery: A Systematic Review and Meta-Analysis of Randomized Controlled Trials. Am. J. Obstet. Gynecol..

[28] Murphy D., Strachan B., Bahl R. (2020). Assisted Vaginal Birth: Green-top Guideline No. 26. BJOG Int. J. Obstet. Gynaecol..

[29] RANZCOG (2020). Instrumental Vaginal Birth; College Statement C-Obs 16.

[30] Ubom A.E., Barnea E.R., DiSimone N., Mueller M., Beyeza-Kashesya J., Nunes I., Nieto-Calvache A.J., Topcu E.G., Malel Z.J., Jacobsson B. (2025). FIGO Good Practice Recommendations: Assisted Vaginal Birth and the Second Stage of Labor. Int. J. Gynecol. Obstet..

[31] Hobson S., Cassell K., Windrim R., Cargill Y. (2019). No. 381-Assisted Vaginal Birth. J. Obstet. Gynaecol. Can..

[32] Jakubowski P., Abele H., Bamberg C., Bogner G., Desery K., Fazelnia C., Hamza A.S., Heihoff-Klose A., Janning L., Kimmich N. (2025). Vaginal-operative Birth: Guideline of the DGGG, OEGGG and SGGG (S2k-Level, AWMF Registry Number 015/023, 11/2023). Geburtshilfe Frauenheilkd..

[33] National Institute for Health and Care Excellence (NICE) (2023). Intrapartum Care (NG235).

[34] Cahill A.G., Raghuraman N., Gandhi M., Kaimal A.J. (2024). First and Second Stage Labor Management: ACOG Clinical Practice Guideline No. 8. Obstet. Gynecol..

[35] Brazilian Federation of Gynecology and Obstetrics Associations (Febrasgo) (2023). Operative Vaginal Delivery.

[36] Malvasi A., Malgieri L.E., Stark M., Di Naro E., Farine D., Baldini G.M., Dellino M., Yassa M., Tinelli A., Vimercati A. (2025). The Contribution of AIDA (Artificial Intelligence Dystocia Algorithm) to Cesarean Section Within Robson Classification Group. J. Imaging.

[37] Malvasi A., Malgieri L.E., Cicinelli E., Vimercati A., Achiron R., Sparić R., D’Amato A., Baldini G.M., Dellino M., Trojano G. (2024). AIDA (Artificial Intelligence Dystocia Algorithm) in Prolonged Dystocic Labor: Focus on Asynclitism Degree. J. Imaging.

[38] Malvasi A., Malgieri L.E., Difonzo T., Achiron R., Tinelli A., Baldini G.M., Vasciaveo L., Beck R., Mappa I., Rizzo G. (2025). Artificial Intelligence Dystocia Algorithm (AIDA) as a Decision Support System in Transverse Fetal Head Position. J. Imaging.

[39] O’Mahony F., Hofmeyr G.J., Menon V. (2010). Choice of instruments for assisted vaginal delivery. Cochrane Database Syst. Rev..

[40] Ismail N.A.M., Saharan W.S.L., Zaleha M.A., Jaafar R., Muhammad J.A., Razi Z.R.M. (2008). Kiwi Omnicup versus Malmstrom Metal Cup in Vacuum Assisted Delivery: A Randomized Comparative Trial. J. Obstet. Gynaecol. Res..

[41] Anteby M., Pinchas-Cohen T., Baruch Y., Lavie A., Maslovitz S., Hiersch L., Yogev Y. (2025). The Impact of Metal Cup Size on Neonatal and Maternal Morbidity in Vacuum-Assisted Deliveries. Int. J. Gynecol. Obstet..

[42] Panelli D.M., Leonard S.A., Joudi N., Judy A.E., Bianco K., Gilbert W.M., Main E.K., El-Sayed Y.Y., Lyell D.J. (2023). Clinical and Physician Factors Associated with Failed Operative Vaginal Delivery. Obstet. Gynecol..

[43] Kane D., Wall E., Malone E., Geary M.P., Malone F., Kent E., McCarthy C.M. (2023). A Retrospective Cohort Study of the Characteristics of Unsuccessful Operative Vaginal Deliveries. Eur. J. Obstet. Gynecol. Reprod. Biol..

[44] Opoku B. (2006). A Review of Vacuum Deliveries at Komfo Anokye Teaching Hospital, Kumasi. Ghana Med. J..

[45] Verhoeven C.J., Nuij C., Janssen-Rolf C.R.M., Schuit E., Bais J.M.J., Oei S.G., Mol B.W.J. (2016). Predictors for Failure of Vacuum-Assisted Vaginal Delivery: A Case-Control Study. Eur. J. Obstet. Gynecol. Reprod. Biol..

[46] Grasch J.L., Venkatesh K.K., Grobman W.A., Silver R.M., Saade G.R., Mercer B., Yee L.M., Scifres C., Parry S., Simhan H.N. (2023). Association of Maternal Body Mass Index with Success and Outcomes of Attempted Operative Vaginal Delivery. Am. J. Obstet. Gynecol. MFM.

[47] McTiernan A.M., Ruprai C.K., Lindow S.W. (2023). Assisted Vaginal Delivery in the Obese Patient. Best Pract. Res. Clin. Obstet. Gynaecol..

[48] Lang Ben Nun E., Sela H.Y., Ioscovich A., Rotem R., Grisaru-Granovsky S., Rottenstreich M. (2022). Epidural Analgesia and Vacuum-Assisted Delivery in Primiparous Women: Maternal and Neonatal Outcomes. J. Matern. Fetal Neonatal Med..

[49] Ben-Haroush A., Melamed N., Kaplan B., Yogev Y. (2007). Predictors of Failed Operative Vaginal Delivery: A Single-Center Experience. Am. J. Obstet. Gynecol..

[50] Sheiner E., Shoham-Vardi I., Silberstein T., Hallak M., Katz M., Mazor M. (2001). Failed Vacuum Extraction. Maternal Risk Factors and Pregnancy Outcome. J. Reprod. Med..

[51] Kabiri D., Lipschuetz M., Cohen S.M., Yagel O., Levitt L., Herzberg S., Ezra Y., Yagel S., Amsalem H. (2019). Vacuum Extraction Failure Is Associated with a Large Head Circumference. J. Matern. Fetal Neonatal Med..

[52] Wanyonyi S.Z., Achila B., Gudu N. (2011). Factors Contributing to Failure of Vacuum Delivery and Associated Maternal/Neonatal Morbidity. Int. J. Gynaecol. Obstet..

[53] Le Brun C., Beucher G., Morello R., Jones F., Lamendour N., Dreyfus M. (2013). Échecs d’extraction par ventouse obstétricale: Facteurs de risque, conséquences maternelles et fœtales. J. Gynécologie Obs. Biol. Reprod..

[54] Sau A., Sau M., Ahmed H., Brown R. (2004). Vacuum Extraction: Is There Any Need to Improve the Current Training in the UK?. Acta Obstet. Gynecol. Scand..

[55] Cotzias C.S., Paterson-Brown S. (1998). Ventouse: Use, Cup Placement and Success. J. Obstet. Gynaecol..

[56] Palatnik A., Grobman W.A., Hellendag M.G., Janetos T.M., Gossett D.R., Miller E.S. (2016). Predictors of Failed Operative Vaginal Delivery in a Contemporary Obstetric Cohort. Obstet. Gynecol..

[57] Rizzo G., Mattioli C., Mappa I., Bitsadze V., Khizroeva J., Makatsariya A., D’Antonio F. (2021). Antepartum Ultrasound Prediction of Failed Vacuum-Assisted Operative Delivery: A Prospective Cohort Study. J. Matern. Fetal Neonatal Med..

[58] Sullivan C., Hayman R. (2008). Instrumental Vaginal Delivery. Obstet. Gynaecol. Reprod. Med..

[59] Hayman R. (2005). Instrumental Vaginal Delivery. Curr. Obstet. Gynaecol..

[60] Cuerva M.J., Espinosa J.Á., Barras S., Gonzalez-Cerron S., Ojeda F., Cortés M. (2020). Which Technique Is Better to Place a Manoeuvrable Vacuum Extractor Cup on the Flexion Point? Vacca vs. Bird Technique. J. Perinat. Med..

[61] Pettersson K., Ajne J., Yousaf K., Sturm D., Westgren M., Ajne G. (2015). Traction Force during Vacuum Extraction: A Prospective Observational Study. BJOG Int. J. Obstet. Gynaecol..

[62] Malvasi A., Barbera A., Di Vagno G., Gimovsky A., Berghella V., Ghi T., Di Renzo G.C., Tinelli A. (2015). Asynclitism: A Literature Review of an Often Forgotten Clinical Condition. J. Matern. Fetal Neonatal Med..

[63] Ghi T., Bellussi F., Azzarone C., Krsmanovic J., Franchi L., Youssef A., Lenzi J., Fantini M.P., Frusca T., Pilu G. (2016). The “Occiput–Spine Angle”: A New Sonographic Index of Fetal Head Deflexion during the First Stage of Labor. Am. J. Obstet. Gynecol..

[64] Iversen J.K., Kahrs B.H., Eggebø T.M. (2021). There Are 4, Not 7, Cardinal Movements in Labor. Am. J. Obstet. Gynecol. MFM.

[65] Iversen J.K., Kahrs B.H., Torkildsen E.A., Eggebø T.M. (2020). Fetal Molding Examined with Transperineal Ultrasound and Associations with Position and Delivery Mode. Am. J. Obstet. Gynecol..

[66] Schiff E., Friedman A., Mashiach S., Hart R., Kaplan B. (2001). A Matched Controlled Study of Kielland’s Forceps for Transverse Arrest of the Fetal Vertex. J. Obstet. Gynaecol..

[67] Muraca G.M., Sabr Y., Lisonkova S., Skoll A., Brant R., Cundiff G.W., Joseph K.S. (2017). Perinatal and Maternal Morbidity and Mortality after Attempted Operative Vaginal Delivery at Midpelvic Station. Can. Med. Assoc. J..

[68] Melamed N., Yogev Y., Steinmetz S., Ben-Haroush A. (2009). What Happens When Vacuum Extraction Fails?. Arch. Gynecol. Obstet..

[69] Chamagne M., Perdriolle-Galet E., Baumann C., Morel O. (2019). Quelle voie d’accouchement choisir après échec d’extraction par ventouse. Gynécologie Obs. Fertil. Sénologie.

[70] Bhide A., Guven M., Prefumo F., Vankalayapati P., Thilaganathan B. (2007). Maternal and Neonatal Outcome after Failed Ventouse Delivery: Comparison of Forceps versus Cesarean Section. J. Matern. Fetal Neonatal Med..

[71] Miot S., Riethmuller D., Deleplancque K., Teffaud O., Martin M., Maillet R., Schaal J.-P. (2004). Césarienne pour échec d’extraction par ventouse obstétricale: Facteurs de risque et conséquences maternelles et néonatales. Gynécologie Obs. Fertil..

[72] Attali E., Reicher L., Many A., Maslovitz S., Gamzu R., Yogev Y. (2022). Pregnancy Outcome after Cesarean Section Following a Failed Vacuum Attempt. J. Matern. Fetal Neonatal Med..

[73] Ahlberg M., Norman M., Hjelmstedt A., Ekéus C. (2016). Risk Factors for Failed Vacuum Extraction and Associated Complications in Term Newborn Infants: A Population-Based Cohort Study. J. Matern. Fetal Neonatal Med..

[74] Hendler I., Kirshenbaum M., Barg M., Kees S., Mazaki-Tovi S., Moran O., Kalter A., Schiff E. (2017). Choosing between Bad, Worse and Worst: What Is the Preferred Mode of Delivery for Failure of the Second Stage of Labor?. J. Matern. Fetal Neonatal Med..

[75] Młodawska M., Młodawski J., Świercz G., Zieliński R. (2022). The Relationship between Nuchal Cord and Adverse Obstetric and Neonatal Outcomes: Retrospective Cohort Study. Pediatr. Rep..

[76] Edgar D.C., Baskett T.F., Young D.C., O’Connell C.M., Fanning C.A. (2012). Neonatal Outcome Following Failed Kiwi OmniCup Vacuum Extraction. J. Obstet. Gynaecol. Can..

[77] Al-Kadri H., Sabr Y., Al-Saif S., Abulaimoun B., Ba’Aqeel H., Saleh A. (2003). Failed Individual and Sequential Instrumental Vaginal Delivery: Contributing Risk Factors and Maternal–Neonatal Complications. Acta Obstet. Gynecol. Scand..

[78] De Jonge E.T., Lindeque B.G. (1991). A Properly Conducted Trial of a Ventouse Can Prevent Unexpected Failure of Instrumental Delivery. South Afr. Med. J. Suid Afr. Tydskr. Geneeskd..

[79] Wong G.Y., Mok Y.M., Wong S.F. (2007). Transabdominal Ultrasound Assessment of the Fetal Head and the Accuracy of Vacuum Cup Application. Int. J. Gynecol. Obstet..

[80] Garcia-Jimenez R., Valero I., Borrero C., Garcia-Mejido J.A., Fernandez-Palacin A., Serrano R., Sainz-Bueno J.A. (2023). Can Intrapartum Ultrasonography Improve the Placement of the Vacuum Cup in Operative Vaginal Deliveries?. Tomography.

[81] Ghi T., Eggebø T., Lees C., Kalache K., Rozenberg P., Youssef A., Salomon L.J., Tutschek B. (2018). ISUOG Practice Guidelines: Intrapartum Ultrasound. Ultrasound Obstet. Gynecol..

[82] Ghi T., Dall’Asta A., Masturzo B., Tassis B., Martinelli M., Volpe N., Prefumo F., Rizzo G., Pilu G., Cariello L. (2018). Randomised Italian Sonography for Occiput POSition Trial Ante Vacuum (R.I.S.POS.T.A.). Ultrasound Obstet. Gynecol..

[83] Goldkamp J., Vricella L., Mostello D., Tomlinson T. (2020). Ultrasound Feedback Training Increases Trainee Accuracy in Vaginal Assessment of Fetal Head Position in Labor. Ultrasound Obstet. Gynecol..

[84] Ramirez Zegarra R., di Pasquo E., Dall’Asta A., Minopoli M., Armano G., Fieni S., Frusca T., Ghi T. (2021). Impact of Ultrasound Guided Training in the Diagnosis of the Fetal Head Position during Labor: A Prospective Observational Study. Eur. J. Obstet. Gynecol. Reprod. Biol..

[85] Cunningham F.G., Leveno K.J., Dashe J.S., Hoffman B.L., Spong C.Y., Casey B.M. (2023). Williams Obstetrics.

[86] Åberg K., Norman M., Pettersson K., Järnbert-Pettersson H., Ekéus C. (2019). Protracted Vacuum Extraction and Neonatal Intracranial Hemorrhage among Infants Born at Term: A Nationwide Case-control Study. Acta Obstet. Gynecol. Scand..

[87] Bahl R., Hotton E., Crofts J., Draycott T. (2024). Assisted Vaginal Birth in 21st Century: Current Practice and New Innovations. Am. J. Obstet. Gynecol..

[88] Mlodawski J., Mlodawska M., Ciebiera M., Cieslik H., Sawicki W. (2023). Repeatability and Reproducibility of Intrapartum Ultrasound Measurements. J. Clin. Med..

